# Prostate Cancer Stem-like Cells Contribute to the Development of Castration-Resistant Prostate Cancer

**DOI:** 10.3390/cancers7040890

**Published:** 2015-11-18

**Authors:** Diane Ojo, Xiaozeng Lin, Nicholas Wong, Yan Gu, Damu Tang

**Affiliations:** 1 Division of Nephrology, Department of Medicine, McMaster University, Hamilton, ON L8N 4A6, Canada; ojod@mcmaster.ca (D.O.); linx36@mcmaster.ca (X.L.); wongnyh@gmail.com (N.W.); yangu@hotmail.ca (Y.G.); 2 Father Sean O’Sullivan Research Institute, Hamilton, ON L8N 4A6, Canada; 3 The Hamilton Center for Kidney Research, St. Joseph’s Hospital, Hamilton, ON L8N 4A6, Canada

**Keywords:** prostate cancer, castration resistant prostate cancer, prostate cancer stem-like cells, androgen receptor, IL6, STAT3

## Abstract

Androgen deprivation therapy (ADT) has been the standard care for patients with advanced prostate cancer (PC) since the 1940s. Although ADT shows clear benefits for many patients, castration-resistant prostate cancer (CRPC) inevitably occurs. In fact, with the two recent FDA-approved second-generation anti-androgens abiraterone and enzalutamide, resistance develops rapidly in patients with CRPC, despite their initial effectiveness. The lack of effective therapeutic solutions towards CRPC largely reflects our limited understanding of the underlying mechanisms responsible for CRPC development. While persistent androgen receptor (AR) signaling under castration levels of serum testosterone (<50 ng/mL) contributes to resistance to ADT, it is also clear that CRPC evolves via complex mechanisms. Nevertheless, the physiological impact of individual mechanisms and whether these mechanisms function in a cohesive manner in promoting CRPC are elusive. In spite of these uncertainties, emerging evidence supports a critical role of prostate cancer stem-like cells (PCSLCs) in stimulating CRPC evolution and resistance to abiraterone and enzalutamide. In this review, we will discuss the recent evidence supporting the involvement of PCSLC in CRPC acquisition as well as the pathways and factors contributing to PCSLC expansion in response to ADT.

## 1. Introduction

Prostate cancer (PC) is a major male health risk in the developed world [1]. The disease develops from high-grade prostatic intra-epithelial neoplasia (HGPIN) to locally invasive carcinoma and to metastatic cancer [2,3]. Ever since the seminal discoveries by Charles Huggins in 1941, which showed surgical castration and estrogen injection led to a significant PC regression as evident by the reduction of serum acid phosphatase in 15 of 21 patients with metastatic PC [4,5], androgen deprivation therapy (ADT) remains the standard of care for patients with advanced PC. While ADT is initially effective in more than 80% of patients, castration-resistant PCs (CRPCs) inevitably develop in men with metastatic PC [6]. Thus, understanding the mechanism mediating CRPC has been a major research focus since. A moderate success was achieved a decade ago when FDA approved docetaxel as a first line-therapy for CRPC. The treatment prolongs the median overall survival in patients with CRPC for approximately 3 months [7,8]; however, resistance always occurs. Accumulative research efforts reveal a contribution of persistent androgen receptor (AR) signaling to CRPC development [9,10]. This knowledge led to the search for better anti-androgens in the last two decades, which resulted in FDA approval of the second-generation steroid synthesis inhibitor abiraterone in 2011, and the AR antagonist enzalutamide in 2012 [11,12]. Both inhibitors conferred survival advantages in men with taxane-resistant CRPC for 4–5 months [11,12]. Unfortunately, resistance followed. Although the underlying mechanisms are still under research, evidence suggests that persistent AR signaling contributes to the development resistance to the second generation anti-androgens. For example, AR mutations and constitutively active AR aberrant splice variants were observed in CRPCs that conferred resistance to abiraterone and enzalutamide [5,13,14]. However, although the concept of persistent AR activity contributing to resistance is in line with the important role of AR in the acquisition of ADT resistance, it is clear that other mechanisms are also involved, including the appearance of AR-negative neuroendocrine PC (NEPC) and AR-pathway independent PC [15]. In this review, we will briefly discuss the contributions of persistent AR signaling to CRPC development and resistance acquisition to abiraterone and enzalutamide, with a specific focus on the emerging role of prostate cancer stem cell-like cells (PCSLCs) in resistance development.

## 2. Persistent AR Signaling Plays a Role in Developing Resistance to Inhibition of AR Signaling

Although our knowledge of the androgen-dependent nature of prostate epithelium was established long before 1941 [4], it was not until this period that Charles Huggins and colleagues made the seminal discovery that prostate cancer has retained this property, *i.e.*, castration and estrogen administration caused regression of metastatic PC [4,5]. Since then, while ADT has continued to evolve, it nonetheless remains the standard of care for patients with advanced PC. However, despite ADT offering initial benefits, CRPC inevitably develops [16]. Although detailed mechanisms governing CRPC development under low levels of serum testosterone (<50 ng/mL) achieved by castration and ADT are largely elusive, accumulative evidence supports the notion that persistent AR signaling is a critical component to this process [9]. This topic has been well reviewed [5,9,17]. We will briefly summarize the evidence to facilitate the following discussion on the involvement of PCSLCs in CRPC development.

Despite castration-resulted reduction of circulating testosterone, the intratumoral levels of androgens in CRPCs are not lower than those in hormone naive tumors [18,19]. In line with these observations, alterations of several steroidogenic enzymes with a collective effect in promoting the intratumoral production of androgens in CRPC and metastatic PCs were observed [20,21]. This knowledge paved the way for the development of abiraterone, a potent CYP17 inhibitor. In addition to inhibiting intratumoral androgen biosynthesis, abiraterone also suppresses the synthesis of adrenal androgens [dehydroepiandrosterone (DHEA) and androstenedione (AD)] [22,23]. This inhibition is significant, as adrenal androgens are abundant in circulation and are efficiently converted by prostate into 5α-dihydrotestosterone (DHT) [24]. Through the inhibition of androgen biosynthesis, abiraterone prolongs the median overall survival by 3.9 and 4.6 months in the respective median follow-up of 12.8 and 20.2 months [11,25] within metastatic CRPC (mCRPC) patients who have been previously treated with docetaxel [11] and by 4.4 months within chemotherapy-naive CRPC patients [26].

Persistent AR signaling in CRPC is also facilitated through AR coactivators. FOXA1 is a pioneer factor that plays a role in AR recruitment to chromatin, a process that is required for AR to transactivate target genes [27,28]. Elevation of FOXA1 occurs in PC and contributes to CRPC via enhancing the AR-chromatin association [29]. Upregulation of three steroid receptor coactivators (SRC) has been reported in PC and is associated with unfavorable prognosis [9]. Amplification of the SRC-2 gene *NCOA2* was reported in 24.3% metastatic PCs and is associated with AR transcription activity [9,30].

A major mechanism leading to persistent AR signaling is AR amplification and mutations. Amplification of the AR gene at Xq11-12 was first reported in 30% (7/23) of CRPCs [31]. A significant AR gene amplification in CRPC compared to hormone naive PC were subsequently confirmed, although the rate varied from 20% (10/49) [32] to 58% [30]. A most recent systemic analysis of whole exome sequencing of 150 mCRPC biopsies revealed 63% of AR gene amplification and mutation in comparison to whole exome sequencing of 440 primary PC tissues [33]. Additionally, multiple aberrant splicing AR variants (AR-Vs) with missing C-terminal ligand-binding domain (LBD) were detected in CRPC [34]. Among them, the AR-V7 (AR1/2/3/CE3 variant) protein was upregulated in CRPC; AR-V7 (AR3) was constitutively active and conferred androgen-independent growth of LNCaP cells *in vitro* and *in vivo* (xenograft tumors) [35,36]. Knockdown of AR-V7 abolished the CRPC phenotype [13]. AR-V7 was detected in 39% and 19% of circulating tumor cells derived from CRPC patients treated with abiraterone and enzalutamide, respectively [14]. Moreover, the expression of AR-V7 was associated with resistance to the second-generation anti-androgens and poor prognosis [14]. Collectively, accumulative evidence demonstrates an important role of persistent AR signaling in promoting CRPC development, and likely the development to abiraterone and enzalutamide resistance as well.

## 3. PCSLCs are a Critical Contributor to Resistance Acquisition to AR Inhibition

Several lines of evidence, as discussed above, support a critical role of persistent AR signaling in CRPC development, including the transition from the paracrine to autocrine dependency on androgens, upregulation of AR coactivators, and the recurrent amplification and mutations in the *AR* gene. More importantly, the functional status of persistent AR signaling in CRPC is demonstrated by the effectiveness of abiraterone and enzalutamide in treating patients with CRPC [11,12].

However, it is also clear that complex mechanisms regulate the resistance to ADT and to the second-generation anti-androgens. Genetically, copy number alterations occur in numerous genomic loci besides AR amplification in metastatic PC [37]. PCs are commonly associated with a large number of chromosomal rearrangements [38]. Complex epigenetic changes arise following PC progression. For example, a significant increase in CpG island methylation correlates with PC progression from benign prostate tissues to carcinomas, and from hormone naïve carcinomas to CRPC [39]. Hypermethylation can be resulted from PC-associated hyperproliferation [40]. The effectiveness of ADT associates with the status of PC evolution. For example, ADT was more efficacious to PCs at early stages [41]; whereas enzalutamide was less effective in treating abiraterone-resistant CRPCs [42,43,44] and vice versa [45]. At the molecular level, AR signaling inhibits some pathways critical for PC evolution, including c-Met and AKT activation [46,47,48]. Taken together, it is clear that mechanisms in addition to persistent AR signaling play a role in the acquired resistance to ADT, abiraterone and enzalutamide. Although these mechanisms are not fully understood, it is evident that they collectively promote CRPC evolution.

### 3.1. Prostate Cancer Stem-Like Cells

Definitive demonstration of the prostate cancer stem cell (PCSC) properties (initiation of tumors from true PCSCs and self-renewal via serial tumor transplantation) remains a challenge for the PC research community. However, accumulative evidence supports the existence of PCSCs or prostate cancer stem-like cells. PCSLCs bearing a specific surface antigen profile (CD44^+^/α_2_β_1_^hi^/CD133^+^) were isolated from primary PC tissues; these cells showed high levels of clonogenic ability [49]. Recent developments suggested that these cells expressed active telomerase activity and increased telomere length [50]. Human PC cells ectopically expressing human telomerase reverse transcriptase (hTERT) displayed the CD44^+^α_2_β_1_^hi^CD133^+^ surface profile and these cells were able to regenerated heterogeneous xenograft tumors representing the primary prostate tumors [51,52]. The CD44^+^ subpopulation isolated from several cultured PC cell lines was more tumorigenic than its isogenic CD44^−^ populations [53]. PCSLCs also expressed a cell surface detoxification pump ABCG2 [54], which may play a role in the cells’ intrinsic resistance to a variety of cytotoxic effects. Lineage-tracking analysis in a mouse model of PC revealed cells with prostate stem cell markers producing PC [55]. At the molecular level, PCSLCs express well-established pluripotency genes, including SOX2, NANOG, and OCT4 [52,56,57] and generally do not express differentiation markers, such as AR and prostate specific antigen (PSA) [51,58].

### 3.2. PCSLC Contributes to CRPC

Multiple lines of evidence support the notion that PCSLCs play important roles in resistance acquisition to anti-androgen therapies. The development of resistance towards ADT, enzalutamide or abiraterone is regulated by PC’s ability to evolve, a trait of PCSLCs. It is commonly recognized that among tumor’s heterogeneous cell populations [59,60,61], cancer stem cells (CSCs) are the units of evolutionary selection [62,63,64]. This is more relevant with respect to therapy-induced resistance. CSCs possess intrinsic resistance to therapy-induced cytotoxicity [65,66] and are able to evolve following the selective pressure derived from treatments [67]. For example, PCSLCs are significantly more resistant than their non-cancer stem cell counterparts towards etoposide- and γ-irradiation-induced DNA damage [68,69]. This plasticity of CSCs in directing tumor recurrence is shared by their tissue stem cell counterparts during tissue regeneration [70,71]. Akin to orchiectomy that leads to robust prostate involution, ADT results in marked PC regression. Both prostate stem cells (PSCs) and PCSLCs are androgen-independent. While PSCs regenerate the prostate, PCSLCs produce recurrent CRPCs [58,72]. The signatures of PCSLCs are associated with poor prognosis [55,73].

In agreement with the above knowledge, experimental evidence also supports an important role of PCSLCs in CRPC development. During prostate development, NE epithelial cells are differentiated from prostate stem cells [74]; and NEPCs are derived from PCSLCs under ADT [75,76]. Additionally, both abiraterone and enzalutamide induce NEPC, an outcome contributing to their resistance [77,78]. NEPC is also induced by docetaxel-based chemotherapy [79,80]. Docetaxel treatment was reported to increase epithelial-mesenchymal transition (EMT) in PC cells and EMT contributed to resistance to docetaxel in PC [81,82]. Inhibition of EMT reduced PC cell’s resistance to docetaxel and PC cells with PCSLC features showed elevated resistance to docetaxel [81]. EMT is a typical feature of PCSLC (see next section for details). Collectively, the induction of NEPC in response to docetaxel and the second generation of anti-androgens (abiraterone and enzalutamide) treatments suggest that PCSLCs can contribute to therapy-resulted NEPC. Furthermore, the transition from androgen-sensitive to castration-resistant phenotype is associated with PCSLC expansion. For example, *in vitro* CRPC derivatives of LNCaP cells are associated with enrichment of CD44^+^ cells and the ability to form spheres under serum-free conditions [83]. CD44^+^ is a common PCSLC marker [53] and sphere formation is a widely used culture condition for CSC and PCSLCs [84]. More importantly, CRPC-LNCaP cells display a dramatically increased tumor initiation capacity in SCID mice [83]. Androgen deprivation upregulates a set of PCSLC markers in LNCaP cell-based xenograft tumors and TRAMP mice, including CD44, aldehyde dehydrogenase (ALDH), p63, sonic hedgehog (SHH), and Sca-1 [85]. In addition, ADT increases CD44^+^ stem-like cells in CRPC tumors compared to hormone-naïve tumors [86]. The concept of PCSLC contributing to CRPC is also consistent with the observation that the lethal form of bone metastases are heterogeneous in AR-positive and -negative cells [87]; the same heterogeneity was also reported in CRPC metastases [88]. The important roles of PCSLCs in inducing resistance to ADT are also in agreement with ADT stimulating the processes and pathways critical to PCSLC development.

### 3.3. ADT Leads to EMT

**Figure 1:**
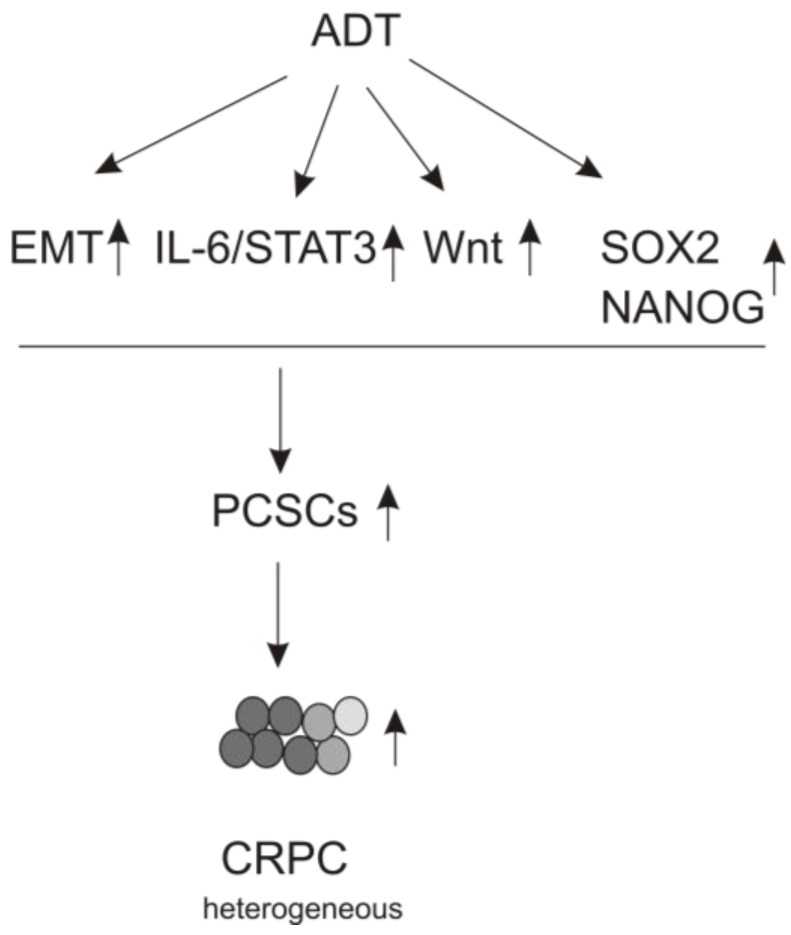
ADT or inhibition of androgen receptor (AR) signaling causes CRPC via induction of PCSLCs. ADT upregulates the indicated events, leading to PCSLC expansion and subsequent CRPC acquisition.

Consistent with EMT being a typical feature of PCSLCs [89], accumulative evidence demonstrates that ADT induces EMT, thereby contributing to CRPC development. ADT stimulates PC cells with EMT and PCSLC features in LuCap35 human prostate explants and tumors from patients [86,90,91]. Those EMT features include downregulation of E-cadherin and upregulation of N-cadherin, vimentin, and a group of E-cadherin repressors (Zeb1/2, Twist, Snail, and Slug). Intriguingly, anti-N-cadherin antibodies dramatically delayed CRPC in a xenograft mouse model [92]. Mechanisms responsible for ADT-induced EMT are complex. A direct negative feedback regulation between AR and Zeb1 has been demonstrated [90], which indicates a direct inhibition of AR on EMT (Figure 1). In tumors from both patients and TRAMP mice, ADT results in EMT. In human PCs, ADT for three months did not clearly induce EMT based on the expression of E-cadherin and N-cadherin, although vimentin was upregulated; in human CRPC on the other hand, significant changes in all these events were observed [86]. In TRAMP mice, castration for 8 weeks resulted in EMT compared to 4 weeks [86]. Collectively, these evidences suggest the involvement of AR inhibition and other alterations for the induction of EMT in response to ADT. This possibility is supported by ADT impacting not only tumors but also their microenvironment [93].

### 3.4. The IL-6/JAK/STAT3 Pathway Plays a Role in ADT-induced PCSLC Evolvement

In line with the alterations of tumor microenvironment contributing to PCSLC expansion under ADT, a large set of evidence reveals a critical role of the IL-6/JAK/STAT3 pathway in PCSLC enrichment under ADT. The IL-6 pathway maintains CSCs/PCSLCs and regulates their plasticity required for cancer evolution, particularly under therapeutic pressure. IL-6 has a key role in systemic inflammation via binding to the IL-6-receptor/gp130 complex, which leads to JAK-derived phosphorylation of STAT3 at tyrosine (Tyr) 705 (pSTAT3) and consequently pSTAT3-mediated transactivation of target genes [94,95]. In accordance with the critical roles of chronic inflammation and immune cells in causing changes in microenvironment during tumorigenesis, IL-6 signaling is required for CSC maintenance in glioma [96,97], lung [98], colon [99], and breast cancer [100,101,102]. Intriguingly, the IL-6 pathway relays the stimuli of chronic low-level inflammation to breast cancer stem cells (BCSCs) [94,100], provides stromal inputs to colon and breast CSCs [103,104], and is instrumental in maintaining breast cancer plasticity by converting non-stem cancer cells to BCSCs [101], thereby promoting resistance to HER2 therapy [105].

The IL-6/JAK/STAT3 pathway stimulates resistance to ADT, abiraterone and enzalutamide. Increases in IL-6 receptor (IL-6R), JAK1, and pSTAT3 in CRPCs were found to associate with poor prognosis [106]. Single nucleotide polymorphisms (SNPs) in the *JAK2* and *STAT3* genes are among the SNPs in the top 9 genes linked to increased risk of PC and PC progression [107]. Elevations of circulating IL-6 were observed in patients with metastatic PC and CRPC [108,109,110], and high levels of serum IL-6 were found to correlate with PC progression and poor prognosis [111]. An upregulation of IL-6/STAT3 signaling occurred in the conversion of premalignant human prostate EPT2-D5 cells to PCSLCs; downregulation of IL-6/STAT3 suppressed the conversion [112]. Elevated STAT3 activity conferred resistance to enzalutamide in LNCaP cell and its knockdown renewed the sensitivity [113]. These results were corroborated with the suppression of recovering prostate cancer stem-like cells (CD44^+^/α_1_β_2_^hi^/CD133^+^) from high-grade primary PC, as well as the prevention of human CRPC tumor growth in mice via inhibition of either IL-6 or STAT3 [114]. Furthermore, reduction of AR signaling resulted in the expansion of PCSLCs that were CD44^+^, α_1_β_2_^hi^, SOX2^+^, and NANOG^+^ with elevated pSTAT3 in xenograft tumors. The expression of pSTAT3 also associates with these stem factors in primary PC. Blocking IL-6 via soluble IL-6R abolished this PCSLC expansion [115]. These results fit well with the observed SOX2 upregulation and EMT following AR inhibition [92,116]. As presented above, clinical, *in vivo*, and *in vitro* evidence all support a critical role of the IL-6/JAK/STAT3 pathway in promoting PCSLC growth under androgen signaling inhibition. PCSLCs then drive PC evolution towards CRPC (Figure 1). This concept is further supported by recent publications demonstrating that ADT and enzalutamide reduced cancer load and promoted PC metastasis by affecting tumor and stroma in a process involving STAT3 [117,118,119,120].

### 3.5. Wnt Signaling Contributing to Resistance to ADT

The Wnt/β-catenin pathway plays a critical role in tissue homeostasis by sustaining the tissue stem/progenitor cells [121]. For example, this pathway maintains the self-renewal of intestinal stem cells; its aberrant activation via inactivation of the adenomatous polyposis coli (APC) tumor suppressor resulted in the upward expansion of undifferentiated progenitors, and thereby contributing to tumorigenesis [122,123]. The Wnt/β-catenin pathway is also utilized by CSCs of other cancer types [124].

Consistent with the contributions of PCSLCs to CRPC development, evidence supports a role of the Wnt/β-catenin pathway in stimulating resistance to ADT. In seven pairs of patient-derived advanced PC with and without ADT treatment for 22 weeks, RNA sequencing identified the Wnt signaling as a top pathway activated in ADT treated tumors [125]. Exome sequencing detected mutations in the Wnt pathway in CRPC but not in hormone native local tumors [126,127]. Collectively, these evidences support a role of the Wnt/β-catenin pathway in stimulating CRPC at least in part through the regulation of PCSLCs (Figure 1).

The Wnt/β-catenin signaling contributes to PCSLCs. Activation of the Wnt pathway via inhibition of GSK3β promoted LNCaP C4-2B and DU145 cell-derived xenograft tumor growth and C4-2B cell-produced bone metastasis. These *in vivo* tumorigenic events were accompanied with an increase in PC cells positive for the well-established PCSLC markers ALDH^+^ and CD133^+^ [128]. In support of these observations, a direct activation of the Wnt pathway using recombinant Wnt3a stimulated nuclear accumulation of β-catenin, sphere formation (*in vitro* assay for self-renewal), and the expression of CD133, CD44, and ABCG2 in LNCaP and LNCaP C4-2 cells [129]. On the other hand, miR-320 was reported to inhibit the Wnt/β-catenin pathway in PC3 and DU145 cells with concurrent reductions in the cell-derived xenograft tumor formation in NOD/SCID mice, as well as the CD44^+^ cell subpopulation, Oct4 and ABCG2 expression [130]. Conversely, in LNCaP and DU145 cell-derived CD44^+^ PCSLC subpopulation, miR-320 expression was substantially reduced along with increases in β-catenin expression. In aggregate, the functional connections of Wnt activation with increases in tumor generation, metastasis, self-renewal, standard PCSC markers (CD133, CD44, and ABCG2), and pluripotency genes (*OCT4* and *NANOG*) strongly support an important role of the Wnt/β-catenin signaling in promoting PCSLCs.

### 3.6. ADT Stimulates PCSLC via Upregulation of Pluripotency Genes

#### 3.6.1. SOX2

SOX2 plays an essential role in maintaining adult stem cells in epithelial tissues [131], and is required to reprogram differentiated cells into inducible pluripotent stem (iPS) cells [132]. Remarkably, this reprogramming process is intrinsically linked to tumorigenesis. The p53 pathway inhibited the reprogramming [133,134,135,136]; and inactivation of p53 empowered SOX2-involved reprogramming of fibroblasts [133]. Epithelial cell-originated cancers apparently explore SOX2’s activities in maintaining their CSCs; SOX2 promotes tumor evolution in multiple tumor types [137], and is required to maintain the self-renewal of breast cancer stem cells (BCSCs) [138].

SOX2 is also critical to sustain the self-renewal status of PCSLCs. Epidermal growth factor receptor (EGFR) maintains the self-renewal of PCSLCs largely through the stimulation of SOX2 expression; knockdown of SOX2 abolished EGFR signaling-initiated PCSLCs [139]. LNCaP cells resistant to androgen deprivation *in vitro* and *in vivo* (xenograft tumors) expressed elevated SOX2 [83]. As well, androgen deprivation induced SOX2 expression in LNCaP cells [140]. AR signaling represses SOX2 in prostate epithelial and PC cells. The SOX2 promoter contains AR-binding elements; via inhibiting AR activation, enzalutamide robustly upregulates SOX2 expression [116]. Furthermore, SOX2 overexpression conferred resistance to androgen removal in LNCaP cells, and facilitated androgen-dependent PC cells in forming xenograft tumors in castrated mice [83,141]. The number of SOX2-positive cells increased following PC progression and metastasis [116]. In essence, these evidences support a model in which ADT or inhibition of AR upregulates SOX2, and SOX2 subsequently stimulates CRPC via PCSLC expansion (Figure 1).

#### 3.6.2. NANOG

In addition to SOX2, NANOG is also an important pluripotency gene [142]. A clear upregulation of NANOG occurs following the progression from benign prostate hyperplasia to high grade PIN (HGPIN) and from HGPIN to PC; NANOG upregulation during PC progression is stimulated by HIF-1α [143]. This is in agreement with the critical role of hypoxia-inducing factors (HIF) in inducing CSCs [144,145]. ADT resulted in PCSLC expressing NANOG in human PC xenografts [72]. GFP-based tracking experiment using the NANOG promoter confirmed NANOG expressing cells to possess PCSLC properties and NANOG overexpression conferred resistance to androgen depletion in LNCaP cells [141]. Furthermore, knockout of *NANOG* and one of its pseudogenes *NANOGP8* decreased DU145 cells’ sphere forming ability *in vitro* and tumorigenesis capacity *in vivo* [146]. DU145 spheroid cells are PCSLC-like cells with elevated tumor initiation ability [84].

## 4. The Contributions of AR Signaling to ADT-induced PCSLC Expansion

**Figure 2:**
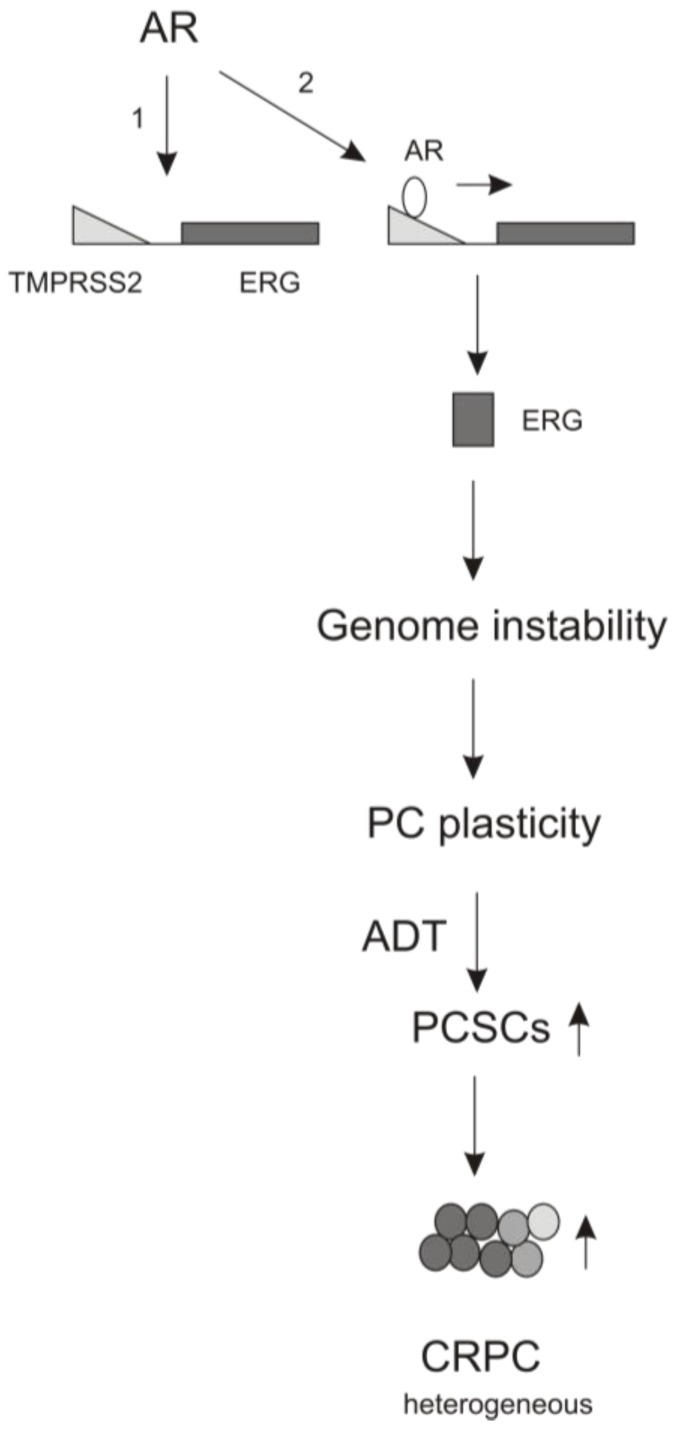
AR signaling indirectly promotes PCSLC via stimulating genome instability. AR signaling brings the AR responsible untranslational (promoter) region of *TMPRSS2* to the proximity of the ERG gene, leading to the production of the fusion gene *TMPRSS2-ERG* (event #1) [149]. AR subsequently transactivates ERG production (event #2), which contributes to genome instability and the resultant PC plasticity. The increased plasticity stimulates PCSLC expansion under ADT.

While AR maintains the normal functions of prostate and promotes prostatic epithelial cell differentiation [6], its roles in PC tumorigenesis are likely different. For example, evidence supports a direct role of AR in promoting the recurrent gene fusion involving the ETS family of transcriptional factors ERG and ETV (*TMPRSS2-ERG* and *TMPRSS2-ETV1*) in PC [147]. It has been observed that androgen induced the recruitment of AR and topoisomerase IIβ (TOP2β) to *TMPRSS2-ERG* genomic breakpoints, which led to the generation of TOP2β-mediated DNA double stranded breaks and the subsequent genomic fusion [148]. Additionally, AR mediates the expression of these fusion genes [149] and ERG subsequently promotes genome instability [147,150]. Collectively, AR may play a role in genome instability, which contributes to PC plasticity and indirectly facilitates PCSLC expansion in response to ADT (Figure 2). This model considers AR’s role in promoting PC progression and supports the clinical observation that ADT is more efficacious to PCs at early stages [41]. This model is also in line with the well-accepted concept that AR signaling is an important contributor to CRPC evolution under ADT.

As it has recently been elegantly reviewed, an important contribution of CSCs to tumor evolution is their derived heterogeneity [151]. Therefore, factors regulating PCSLCs should be heterogeneously expressed in PC. In support of this concept, an examination of 59 hormone naive and 20 hormone refractory (CRPC) tumors using immunohistochemistry demonstrated a heterogeneous pattern of AR expression in both hormone naive PC and CRPC with approximately 25% of tumor cells being AR negative [152]. Similar observations were also reported by others [153,154]. The AR negative status is likely attributed to hypermethylation in the promoter regions of the *AR* gene. It has been reported that 20% of regions in each of 10 primary PC samples and 28% of areas in individuals of 14 CRPC tissues displayed hypermethylation in the 5′ regulatory region of the *AR* gene [155]. Intratumoral heterogeneity in AR also occurs in mCRPC. This heterogeneity was observed at the genomic level; in an effort of whole exome sequencing of 150 mCPRC samples, two functional AR mutations (T878A and Q903H) were detected in the same mCRPC sample [33]. Intratumoral AR heterogeneity in mCRPC has been clearly demonstrated at the protein level [88]. Immunohistochemistry staining of multiple markers, including AR and PSA, in 30 rapid autopsies of mCRPC revealed variations of AR^+^ cells from 0.2% to 87% among individual mCRPC tissues with 31% of tumors containing >50% of AR^+^ cells and 41% of mCRPCs displaying <10% of AR^+^ cells [88].

The observed intratumoral AR heterogeneity suggests an interesting possibility that AR may function differently under different cellular context. Despite PSA being a well-demonstrated AR target, AR^+^ cells might not always translate into PSA^+^ tumor cells. For instance, among the 30 rapid autopsy mCRPC tissues, low percentages of AR^+^ tumors could express high percentage of PSA^+^ cells (0.7% of AR^+^ *versus*/*vs*. 83.5% of PSA^+^; 3.6% of AR^+^ *vs*. 79.6% of PSA^+^) and vice versa (87% of AR^+^ *vs*. 8.5% of PSA^+^; 32.9% of AR^+^ *vs*. 3.3% of PSA^+^) [88]. The existence of AR^+^;PSA^−^ and AR^−^;PSA^+^ tumor cells in both primary and CRPC samples has been recently confirmed [156]. While the majority of the 63% of 150 mCRPC samples with alterations in the *AR* gene did not contain mutations in other components of the AR pathway, like FOX1A and NCOR1/2, there were mCRPC tissues with co-alterations in AR and FOXA1, NCOR1, or NCOR2 [33]. Furthermore, it is well-established that AR signaling is required for LNCaP cell proliferation [157]. However, stable expression of a constitutively active AR mutant in PC3 cells (AR-negative cells) reduced the proliferation to 35% of those without ectopic AR [158].

In support of the above possibility, limited evidence suggests intratumoral AR heterogeneity being associated with the kinetic of CRPC development. For example, in a quantification of AR staining intensity in 200 nuclei for each of the 17 CRPC samples, variations of AR staining were significantly higher in individuals with a rapid kinetics of cancer progression under ADT (*n* = 8, time to progression <20 months) than those with progression time >20 months (*n* = 9) [159]. Collectively, the above discussions support a scenario that AR may contribute to ADT-caused PCSLC.

Indeed, evidence suggests a direct role of AR in facilitating ADT-induced PCSLC expansion. Crosstalk between the Wnt/β-catenin pathway and AR has been demonstrated to enhance CRPC [160,161]. AR binds β-catenin in the nucleus of CRPC and contributes to AR’s transcriptional activities [160,161]. AR is able to transactivate the *NANOG* gene via association with the gene’s promoter [162]. Ectopic expression of AR and AR-V7 in LNCaP and AR-negative DU145 cells enhances EMT, while overexpression of AR-V7 in LNCaP, DU145, and PC3 cells also promotes PCSLC [163]. However, whether these EMT/PCSLC-stimulating activities are attributable to AR transcriptional activity remains unclear, as DHT (dihydrotestosterone) inhibits AR and AR3 (AR-V7) expression and reverses the EMT/PCSLC processes in LNCaP C4-2 and VCaP cells [163]. Nonetheless, these observations suggest a new activity of AR in promoting PCSLC.

## 5. Perspectives

**Figure 3:**
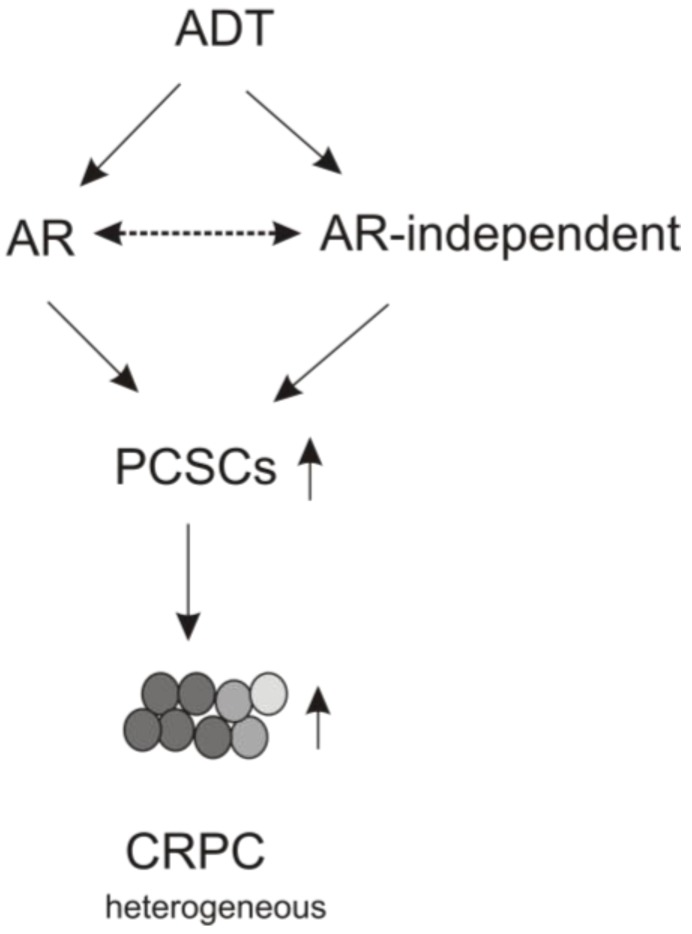
A model describes the contributions of AR-involved and -independent processes to promote PCSLC expansion under ADT. The two-directional dashed arrows indicate potential crosstalk between the two processes.

Despite intensive research for mechanisms leading to CRPC in the past few decades, our understanding remains limited. Nonetheless, it has been noted that persistent AR signaling under ADT levels of serum testosterone (<50 ng/mL) clearly contributes to CRPC, as evident by the short-term survival advantage offered by abiraterone and enzalutamide in CRPC patients [11,12]. However, it is equally clear that AR signaling is not the only contributor and thus, targeting AR alone is unlikely to eliminate CRPC. ADT causes complex alterations within tumors in terms of factors and pathways as well as its epigenetics and genetics. As well, ADT also affects tumor microenvironment. These complex alterations might be particularly relevant to abiraterone and enzalutamide-caused resistance. While resistance to ADT develops in a medium of 12–18 months [6], resistance to abiraterone and enzalutamide occurs within 5.6 and 8.8 months, respectively [11,12]. One strong support for a critical role of AR signaling in resistance acquisition to the new anti-androgens is the involving AR-V7 or AR3, a constitutively active AR mutant [5]. In view of AR-V7 overexpression promotes PCSLC in both AR-positive and -negative PC cells [163], it calls for precaution whether PCSLC expansion should be given an equal emphasis to persistent AR signaling (transcriptional activity) in developing resistance to ADT and the second generation anti-androgens. Furthermore, the intratumoral heterogeneity in AR expression is likely associated with different AR function, which could contribute to PCSLC expansion in response to ADT. With this consideration, it is tempting to propose a model in which ADT results in AR-involved and AR-independent enhancement of PCSLC expansion and the subsequent CRPC acquisition (Figure 3). 

It might be crosstalk between both processes (Figure 3) that explains the situations in which AR is able to suppress EMT and SOX2-mediated PCSLC expansion on one hand (Figure 1) [90,116] while enhancing PCSLC on the other [163] in response to ADT. A critical event in this regard is PCSLC-associated plasticity, *i.e.*, how ADT, abiraterone, and enzalutamide stimulate PCSLC expansion and by what mechanisms PCSLCs drive resistance to the cytotoxic effects imposed by these approaches. Numerous factors and pathways are able to facilitate CRPC acquisition, including AR, PI3K/AKT/PTEN, HIF, IGF-1, Wnt, IL-6, SOX2, NANOG, and many others [5]. It is possible that PCSLC expansion may provide a platform to integrate individual molecular factors/pathways in stimulating CRPC development. The IL-6/JAK/STAT3 pathway is appealing, as it links tumor-derived signaling and tumor microenvironment-initiated signaling. The very recent demonstration of AR-mediated EMT and PCSLC (by AR-V7) is intriguing, although this needs to be further consolidated with studies involving more physiological systems. This is because the development fits well with our knowledge that AR and PCSLCs are important for PC progression, including CRPC evolution. With this in mind, is it possible that AR can facilitate PCSLC expansion independent of its transcriptional activity? This possibility may relieve the conflict between androgen-AR derived transcription activity in mediating prostate epithelial cell differentiation (or PC maintenance) and AR-contributed (transcription-independent) function in PC evolution. Regardless of the specific mechanisms responsible for PCSLC-contributed CRPC development, it is certain that this area shows great clinical potential and is worthy of more research efforts.
